# The COVID-19 mRNA BNT163b2 Vaccine Was Well Tolerated and Highly Immunogenic in Young Adults in Long Follow-Up after Haematopoietic Stem Cell Transplantation

**DOI:** 10.3390/vaccines9101209

**Published:** 2021-10-19

**Authors:** Agnieszka Matkowska-Kocjan, Joanna Owoc-Lempach, Joanna Chruszcz, Edwin Kuźnik, Filip Szenborn, Lidia Jurczenko, Marta Wójcik, Dorota Banyś, Leszek Szenborn, Marek Ussowicz

**Affiliations:** 1 Department and Clinic of Pediatric Infectious Diseases, Wroclaw Medical University, 50-368 Wrocław, Poland; joanna.chruszcz@umed.wroc.pl (J.C.); marta.wojcik@umed.wroc.pl (M.W.); dorota.banys@umed.wroc.pl (D.B.); leszek.szenborn@umed.wroc.pl (L.S.); 2 Department and Clinic of Paediatric Oncology, Haematology and Bone Marrow Transplantation, Wroclaw Medical University, 50-556 Wrocław, Poland; joanna.owoc-lempach@umed.wroc.pl (J.O.-L.); lidia.jurczenko@student.umed.wroc.pl (L.J.); marek.ussowicz@umed.wroc.pl (M.U.); 3 Department of Angiology, Hypertension and Diabetology, Wroclaw Medical University, 50-529 Wrocław, Poland; edvin@op.pl; 4 Faculty of Electronics, Wroclaw University of Science and Technology, 50-370 Wrocław, Poland; filipyoyo@gmail.com

**Keywords:** COVID-19, vaccinations, HSCT

## Abstract

Sixty five patients (18–31 years) who had received allogeneic haematopoietic stem cell transplantation (3–27 years from HSCT) were evaluated for the tolerance and immunogenicity of the COVID-19 mRNA BNT163b2 vaccine. Methods: Patients were vaccinated with two doses at 5 weeks interval. After each dose, patients completed a survey concerning adverse events (AE) and anti-SARS-CoV-2 IgG antibodies were measured before the first vaccine dose (1stVD) and 14–21 days after the second dose (2ndVD). AE reported after 1stVD and 2ndVD, respectively were: fever 0%, 1.7%; fatigue 15.4%, 25.8%; headache 15.4%, 24.1%; chills 6.1%, 12.0%; muscle pain 15.4%, 24.1%; joint pain 3.0%, 6.9%; nausea 6.1%, 6.9%; pain at injection site 30.7%, 34.4%; swelling 3.0%, 10.3%; redness 0, 3.4%; pruritus 0, 5.2%; and axillary lymphadenopathy 3.0%, 1.7%. After 2ndVD, 96.5% patients were positive for anti-SARS-CoV-2 (GMC 3290.94 BAU/mL). No correlation presented between the antibody titer and symptoms of chronic Graft-versus-Host disease, total IgG, lymphocyte CD4+, or AE. Significantly higher titers were observed in COVID-19 convalescents, and inverse correlation (R^2^ = −0.0925, *p* = 0.02) between the time from HSCT and titers after 2ndVD was present. Conclusions: The young adults after HSCT tolerate the COVID-19 mRNA vaccine well and show immunologic response.

## 1. Introduction

Coronavirus disease 2019 (COVID-19) caused by the new β-coronavirus severe acute respiratory syndrome coronavirus 2 (SARS-CoV-2) currently constitutes the leading and overwhelming health issue worldwide. By the date of submission of this article, 241,575,235 COVID-19 cases and 4,916,179 related deaths had been confirmed worldwide [1].

The development of safe and effective COVID-19 vaccines was, without any doubts, the biggest achievement of the 2020 pandemic year. Nevertheless, there are still many people who are hesitant of being vaccinated—including patients with significant medical history, such as bone marrow transplantation [2].

At the end of December 2020, the mRNA COVID-19 vaccines were introduced into public use in Poland. The initial clinical trials that preceded the worldwide use of these vaccines were conducted mostly among generally healthy, non-immunocompromised individuals [3]. Nevertheless, due to extreme risk connected with SARS-CoV-2 infection in patients with coexisting morbidities, the COVID-19 vaccines were highly recommended to all people with chronic diseases, among them patients after haematopoietic stem cell or solid organ transplantation [4]. At the beginning of the vaccine’s use, there were very limited data on the safety and immunogenicity among immunosuppressed or transplant patients, but the vaccines were used despite this as the benefit versus risk ratio in the middle of the COVID-19 pandemic was significantly on the benefit side [5]. In Poland, hematopoietic stem cell transplantation (HSCT) patients were vaccinated as one of the priority groups. All HSCT patients of the age >18 years have had access to the mRNA vaccines since 10th March 2021 due to government regulations, whereas the general population at this time had access to the vaccine depending on age—in March 2021 only people >60 years of age could have been vaccinated, due to shortages in the vaccine supply. As, at this time, there was almost no published data regarding the COVID-19 vaccines safety and tolerance as well as immunogenicity in HSCT patients, we decided to focus on this issue while vaccinating the young adults after HSCT with mRNA BNT163b2 (Comirnaty, Pfizer/Biontech, Mainz, Germany) vaccine.

## 2. Methods

Sixty five young adults who had undergone allogeneic HSCT were included in the prospective observative study. All the patients were transplanted as children, and were still regularly controlled in the largest paediatric bone marrow transplantation centre in Poland. They were vaccinated with mRNA Comirnaty (Pfizer/Biontech) vaccine—2 doses at 5 weeks interval (the shortest available interval at this time—based on limited vaccine supply, Polish government decided to extend the interval between the mRNA vaccine doses—from original 3 weeks to at least 5 weeks). The primary objective was to assess the prevalence and severity of self-reported adverse effects, and the secondary objective the immunogenicity of the vaccine.

All patients were asked to fill in an electronic survey every evening for the first 7 days after each dose of the vaccine, then once per week in the time between the vaccine doses and for 3 weeks after the second dose. The reminders about the survey were sent every day at 6 pm by short message (mobile phones) or by e-mail. Each reminder contained a personalized link to the online survey, which was part of a dedicated web application designed for collecting data. The patients were asked about the presence of systemic symptoms: fatigue, headache, chills, new or worsening muscle pain, new or worsening joint pain, vomiting/nausea, elevated body temperature (37.2–37.9 °C or ≥38.0 °C), and the presence of local reactions: pain, redness, swelling, pruritus at the injection site, and the presence of axillary lymphadenopathy. When a symptom was reported, the patients were asked to define the severity of the symptom. If the patient did not respond to the survey on time, the investigator contacted the patient and asked them to fill in the data.

The reported adverse events (AE) were classified and graded according to the Common Terminology Criteria for Adverse Events (CTCAE) [6].

**Figure 1:**
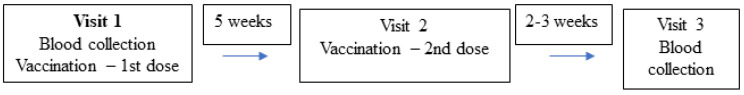
The scheme of the patient’s visits.

The serum sample for anti-SARS-CoV-2 IgG antibodies analysis was collected on the day of the first vaccine dose (before vaccination procedure) in all the patients and 14–21 days after the second dose of the vaccine in the patients who completed the study. The scheme is presented in Figure 1. The sera were tested with the Anti-SARS-CoV-2 QuantiVac ELISA IgG test (Euroimmun/PerkinElmer subsidiary, Waltham, MA, USA). The positive antibodies titer was defined as ≥35.2 BAU/mL.

### Statistical Analysis

Comparisons of the post-vaccination anti-SARS-CoV-2 antibody concentrations between different groups were performed using Mann–Whitney test. The relations between anti-SARS-CoV2 antibody concentration and age, time since HSCT, or time since stopping of immunosuppression were studied with linear regression analysis. Statistical analysis and data presentation were performed with the computer software GraphPad Prism 6.07 (GraphPad Software, La Jolla, CA, USA) and Statistica 13.0 (Statsoft/Dell, Palo Alto, CA, USA). A *p* value less than 0.05 was considered significant.

Basic data regarding each patient was also collected: sex, age, diagnosis, time from allo-HSCT, presence of chronic graft-versus-host disease (cGvHD), immunosuppression use, and history of COVID-19 diagnosis. The patient characteristics are presented in Table 1.

## 3. Results

### 3.1. Adverse Events

A total of 65 patients received the first dose of the vaccine and filled in the survey after the first dose; 59 patients received the second dose during the study time, and 58 answered the survey after the second dose. Six patients could not receive the second dose on time and were withdrawn from the analysis concerning the second vaccine dose. Reasons for withdrawal were: infection with SARS-CoV-2 within the first week after the first dose of vaccine—one patient; symptoms of unidentified upper respiratory tract infection—three patients; quarantine after contact with SARS-CoV-2—one patient. One patient withdrew consent to participate in the study due to personal reasons. No one was excluded due to side effects of the vaccine.

Among 58 patients who answered the survey the following number complained about at least one adverse symptom after vaccination: 23 after both doses, 4 after the first dose only, 8 after the second dose only, and 23 neither after the first nor after the second dose. The most commonly reported systemic symptoms were: headache, fatigue, muscle pain, and the most commonly reported local symptom was pain at the injection site. The detailed results are presented in Table 2. None of our patients who presented the AE reported the severity greater than grade 2 CTCAE. Most symptoms fulfilled the definition of grade 1 CTCAE (mild symptoms; clinical or diagnostic observations only; intervention not indicated); some could be classified as grade 2 CTCAE (moderate; minimal, local, or noninvasive intervention indicated; limiting age-appropriate instrumental activities of daily living). There was one case of diagnostic hospitalization within 2 weeks of the first dose of the vaccine—one of the study participants started to experience mild subjective dyspnoea on the day of vaccination and it persisted for the next 2 weeks. She was admitted to the hospital for diagnostics (1 day). Arterial oxygen saturation, arterial blood gas test, coagulation parameters, electrocardiography, echocardiography, C-reactive protein (CRP), complete blood counts, troponin, and chest imaging were within normal limits. No evidence of organic disease was demonstrated, and finally psychogenic origin of presented dyspnoea was diagnosed. This patient did not present this symptom after the second dose of the vaccine.

There was no relationship between the presence of systemic or local adverse events or the length of their occurrence and concentration of the IgG before vaccination, age, presence of cGvHD symptoms, time from HSCT, or time from the end of immunosuppressive treatment.

### 3.2. Immune Response

A total of 65 patients were tested for anti-SARS-CoV-2 IgG antibodies before the first dose of the vaccine; 57 patients were tested again after the second dose of the vaccine (seven patients were not tested for the second time due to study withdrawal, one patient vaccinated for the second time did not come back to the centre for the second blood collection).

**Figure 2:**
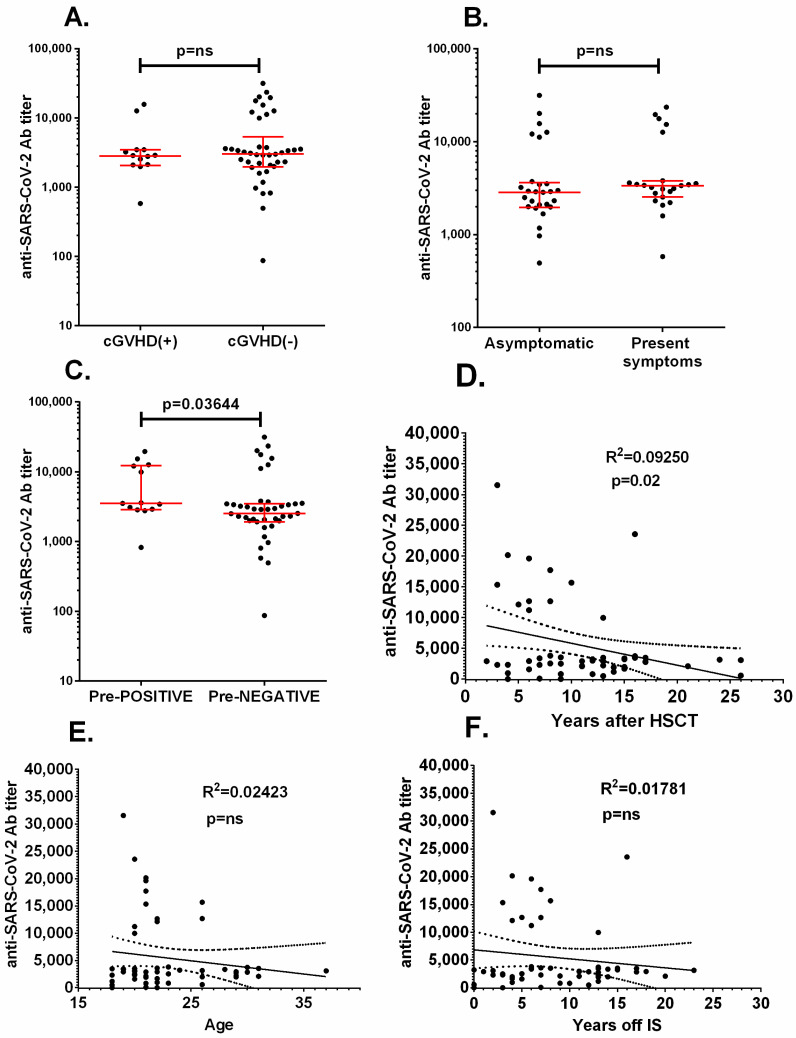
Comparison of anti-SARS-CoV-2 specific antibodies titers in: (**A**) patients with chronic Graft-versus-Host Disease (cGvHD) or without cGvHD; (**B**) patients showing vaccination associated symptoms or asymptomatic; (**C**) patients positive before first dose (Pre-POSITIVE) versus negative (Pre-NEGATIVE). The linear regression analysis of (**D**) anti-SARS-CoV-2 specific antibodies titers and time since HSCT, (**E**) age of the patient, and (**F**) years without immunosuppression. Ns—non significant.

After the second dose of the vaccine, 55/57 patients (96.5%) were positive for the presence of anti-SARS-CoV-2 antibodies. The geometric mean concentration (GMC) of the antibodies 2–3 weeks after the second dose was 3290.94 BAU/mL (range 86.91–31,532.48 BAU/mL). Two patients (3.5%) were negative for the presence of anti-SARS-CoV-2 antibodies after the second dose of vaccine. There was no correlation between the antibody titer after the second dose of the vaccine and presence of cGvHD symptoms, level of total IgG, lymphocyte CD4+ count, or presence of vaccination-associated reactions (Figure 2A,B). Before the first vaccination, 14 patients were positive for the presence of anti-SARS-CoV-2 antibodies (SARS-CoV-2 natural infection convalescents). The GMC of the antibodies before vaccination in positive patients was 90.86 BAU/mL (range 36–341 BAU/mL). Only five patients were aware of the previous SARS-CoV-2 infection (they were tested positive PCR for SARS-CoV-2 at some point during the COVID-19 pandemic). After the second dose, significantly higher titers of antibodies were observed in this group in comparison with primary negative patients (Figure 2C). There was inverse correlation (R^2^ = −0.0925, *p* = 0.02) between the time from HSCT and the concentration of the anti-SARS-CoV-2 antibodies after full vaccination (Figure 2D). There were no such correlations between the age of the patients or the time from the end of immunosuppressive treatment and the concentrations of the anti-SARS-CoV-2 specific antibodies (Figure 2E,F).

## 4. Discussion

The results of patients after HSCT have not been directly compared with the control healthy group for various reasons. Firstly, at the time of data collection (March 2021) healthy young adults had no access to COVID-19 vaccines in Poland due to serious vaccine shortages (only >60 years old and a few high-risk groups were being vaccinated at this time). The priority was to vaccinate patients after bone marrow transplantation as soon as possible to protect them from SARS-CoV-2 infection; delaying the study to a time when a healthy control group could be created was unethical. Secondly, a comparison of people vaccinated in March/April 2021 with healthy young adults who were allowed to receive the first vaccine dose in late May 2021 would be burdened with error caused by COVID-19 epidemy dynamics. In March/April 2021 a massive “wave” of COVID-19 was devastating the Polish population, reaching one of the highest number of COVID-19 cases in Europe [7]. Many people were exposed to the natural SARS-CoV-2 infection which could have had an impact on the study results if the control group was recruited a few weeks later than the HSCT patients.

There is available data of Comirnaty tolerance in the general population [8]. According to Centers for Disease Control and Prevention (CDC) data the percentages of people affected by side effects after the first and the second vaccine dose were, respectively: redness 4.5% and 7.25%; swelling 5.8% and 7.5%; pain 83.1% and 66,1%; fever 3.7% and 15.8%; fatigue 47.4% and 59.4%; headache 41.9% and 51.7%; chills 14.0% and 35.1%; muscle pain 21.3% and 37.3%; joint pain 11% and 21.9% [9]. The comparison of our study results with this data may suggest that young adults after bone marrow transplantation tolerate mRNA COVID-19 vaccine no worse than the general population, which was also seen by other authors [10]. However, this comparison should be taken with caution considering significantly smaller number of patients involved in this study in comparison with reports describing general population.

The results of anti-SARS-CoV-2 antibodies response in the study group are encouraging. There were only 2/58 patients who did not produce specific antibodies after the second dose of the vaccine. One of them (female, 22 years; 9 years after allo-HSCT due to acute myeloid leukaemia) was still receiving cyclosporine A and corticosteroids due to severe chronic GvHD, and the other patient (female, 18 years, 4 years after HSCT due to acute myeloid leukaemia) had persistent deep hypogammaglobulinemia, which may explain the poor serological answer to the vaccine. The rest of the patients presented high concentrations of anti-SARS-CoV-2 IgG antibodies 2–3 weeks after the vaccination completion.

The small subgroup of patients who initially presented positive for anti-SARS-CoV-2 antibodies responded to vaccine without significant sequelae. It shows that the vaccination can be safely recommended to COVID-19 convalescents, as detection of postinfectious anti-SARS-CoV-2 antibodies is not synonymous with durable immunity.

It must be stressed that there is still no agreed correlate of protection against SARS-CoV-2 infection. At the time of this publication there is some evidence that the use of post-immunization antibody titers as the basis for establishing a correlate of protection for COVID-19 vaccines may be justified [11,12]. However, still it is not known if there is any “cut-off” level of antibody concentration that is protective against COVID-19 and what exactly this level is. Some studies have shown the correlation between specific threshold of anti-SARS-CoV-2 antibodies that correspond to virus neutralisation in in vitro plaque reduction neutralisation tests, but the clinical significancy of these findings is still unknown [12]. When this study was performed, it was recommended that all the patients after HSCT should be vaccinated against COVID-19 in the same schedule as the rest of population (two doses). In August/September 2021, for those patients who were less than 2 years after HSCT procedure or who were still receiving immunosuppressive treatment, a third dose of COVID-19 vaccine was recommended by United States Food and Drug Administration (FDA) [13]. However, for many other vaccines, the vaccination schedules for HSCT patients differ from the schedules recommended for the general population, irrespective of the time that has passed after HSCT. Post-HSCT patients are recommended to have more doses of some vaccines than healthy people. For example, an adult patient after HSCT is recommended to be vaccinated with three doses of pneumococcal conjugate vaccine (PCV 13) in 1–2 months intervals (and it does not depend on the time interval from HSCT), whereas a schedule for a healthy adult contains only one dose of PCV 13 [14]. The patients in our study were transplanted quite a long time ago (the median time after HSCT was 10 years). As most of them produced high concentrations of specific anti-SARS-CoV-2 antibodies after vaccination, this may point to the conclusion that the patients in such a long follow-up after HSCT probably do not require additional COVID-19 vaccine doses in the basic vaccination schedule, and should be vaccinated in the same manner as the general population. However, it is not known whether there will be a need of booster doses in the future—nor is it presently known for the general population. The other fact is that the recommendations concerning revaccination after HSCT were usually based on the data received from patients in the first years after HSCT. It is not really known if a person who was transplanted many years ago, who is in a good immune condition and has never been vaccinated, still requires more doses of some vaccines in a basic schedule than a completely healthy person. Chevallier et al. already showed that the patients after HSCT who were vaccinated with COVID-19 mRNA vaccine in the first 2 years after transplantation showed poorer immunologic answer after the first dose of the vaccine than patients who were transplanted a longer time ago [15]. Additionally, Redjoul et al. showed only 78% seropositivity after a second dose in patients vaccinated at a median of 23 months after allogeneic HSCT, which is less than presented in our study [16]. This also may lead to the hypothesis that the patients in long follow up after HSCT procedure may not require so intensive revaccination schedules as is suggested in the general recommendations for HSCT patients.

Our results differ from the observations of solid organ transplant (SOT) patients in whom the immunological answer after the COVID-19 vaccination is severely impaired [17]. SOT recipients usually require long lasting immunosuppressive treatment, that cannot be stopped or suspended. There is more and more evidence that, as a rule, SOT patients should receive the third dose of the COVID-19 vaccine in the basic vaccine schedule [18,19]. Our study shows that the third dose in the basic schedule is probably not necessary in most HSCT patients transplanted more than two ago. However, it is not yet known if there is a need of a booster dose in some time interval (several months or years) after the basic two-dose vaccination schedule. Further studies are needed to evaluate the persistence of immunity after basic vaccination schedule in HSCT patients and to assess the actual clinical efficacy of COVID-19 vaccines.

We discovered that the concentration of anti-SARS-CoV-2 antibodies reversely correlated with the time from the HSCT, but not with the age of the patients, or time from immunosuppression discontinuation. Very weak relationship between time from HSCT and postvaccination specific antibodies titers argues against significant biological effect and does not undermine vaccine efficacy. This fact can reflect a radical change that was achieved in transplantation techniques (reduction in total body irradiation- or busulfan-based regimens) in the last two decades, and widespread use of lower-intensity pre-transplant conditioning protocols with lower incidence of long-term sequelae.

In contrast, the effect of age on vaccine response was noted in case of general population, but data were collected in people aged 60–80 years, which is beyond the upper age limit in our study [20,21].

The age at HSCT was proven to adversely affect the thymic output in children due to thymic involution, and it can be seen as a factor resulting in lower immune response among transplant survivors, but further studies are needed [22].

## 5. Conclusions

The young adults in long follow up after hematopoietic stem cell transplantation tolerate the COVID-19 vaccine well. The immunologic response after two doses of mRNA Comirnaty vaccine is satisfactory in most patients. The data confirming immunogenicity in the immunocompromised population suggest that preventive strategy with mRNA vaccines is feasible in this population, but longer observation and monitoring of real-world effectiveness are warranted.

## Figures and Tables

**Table 1:** Basic information about the patients.

Sex	Male 39, Female 26
Age	18–31 years (median 21)
Diagnosis	acute lymphoblastic leukemia—25
	severe aplastic anemia—9
	chronic myeloid leukemia—9
	myelodysplastic syndrome—6
	acute myeloblastic lekemia—5
	Fanconi Anemia—3
	severe combined immunodeficiency—3
	common variable immunodeficiency—1
	primitive neuroectodermal tumor—1
	metachromatic leukodystrophy—1
	Blackfan-Diamond anemia—1
	Hodgkin lymphoma—1
Time from allo-HSCT	3–27 years (median 10.5)
Symptoms of cGvHD at vaccination	present in 15 patients
Time from immunosuppression end	1–20 years (median 7.5), 4 patients still receiving immunosuppressive treatment
Total IgG levels	within normal range in 48 patients, below normal range in 17 patients
T CD 4 lymphocyte count	within normal range in 57 patients, below normal range in 8 patients

**Table 2:** Adverse events after each vaccine dose.

Symptom	Patients Presenting the Symptom after 1st Dose of Vaccine (*n* = 65)	Median (Range) (Days)	Patients Presenting the Symptom after 2nd Dose of Vaccine (*n* = 58)	Median (Range) (Days)	Patients in Whom the Symptom Was Present after Both Doses
Temp. 37.2–37.9 °C	3 (4.6%)	2 (2–2)	4 (6.9%)	1 (1–1)	1
Temp. 38.0–38.4 °C	0		1 (1.7%)	3	0
Fatigue	10 (15.4%)	2 (1–3)	15 (25.8%)	2 (1–3)	6
Headache	10 (15.4%)	2 (1–7)	14 (24.1%)	2 (1–7)	6
Chills	4 (6.1%)	1 (1–3)	7 (12.0%)	2 (1–3)	1
New or worsening muscle pain	10 (15.4%)	2 (1–4)	14 (24.1%)	2 (1–5)	6
New or worsening joint pain	2 (3.0%)	2 (2–2)	4 (6.9%)	2 (1–3)	1
Vomiting/Nausea	4 (6.1%)	2 (1–2)	4 (6.9%)	2 (2–2)	2
Pain at injection site	20 (30.7%)	2 (1–4)	20 (34.4%)	2 (1–5)	15
Swelling at the injection site	2 (3.0%)	2–3	6 (10.3%)	2 (1–5)	2
Redness at the injection site	0		2 (3.4%)	2 (2–2)	0
Pruritus at the injection site	0		3 (5.2%)	1 (1–5)	0
Axillary lymphadenopathy	2 (3.0%)	2 (2–2)	1 (1.7%)	2	0

## Data Availability

The data presented in this study is available on request from the corresponding author.
